# Supplementary material for: Scratch-AID, a deep learning-based system for automatic detection of mouse scratching behavior with high accuracy
Source: eLife. 2022 Dec 8;11:e84042. doi: 10.7554/eLife.84042 (PMC9762698; doi:10.7554/eLife.84042)
Supplement: Supplementary file 1. [file elife-84042-supp1.docx]

**Supplementary file 1. Mouse information used in the recording of the training and test videos**

| **Video No.** | **Mouse No.** | **Injection site (nape)** | **Sex** | **Weight** | **Age** |
| --- | --- | --- | --- | --- | --- |
| V1 | M1 | Left 1 | F | 20.6g | 8-12 week |
| V2 |  | Left 2 |  |  |  |
| V3 |  | Right 1 |  |  |  |
| V4 |  | Right 2 |  |  |  |
| V5 | M2 | Left 1 | F | 21.1g |  |
| V6 |  | Left 2 |  |  |  |
| V7 |  | Right 1 |  |  |  |
| V8 |  | Right 2 |  |  |  |
| V9 | M3 | Left 1 | F | 23.3g |  |
| V10 |  | Left 2 |  |  |  |
| V11 |  | Right 1 |  |  |  |
| V12 |  | Right 2 |  |  |  |
| V13 | M4 | Left 1 | F | 21.2g |  |
| V14 |  | Left 2 |  |  |  |
| V15 |  | Right 1 |  |  |  |
| V16 |  | Right 2 |  |  |  |
| V17 | M5 | Left 1 | F | 21.4g |  |
| V18 |  | Left 2 |  |  |  |
| V19 |  | Right 1 |  |  |  |
| V20 |  | Right 2 |  |  |  |
| V21 | M6 | Left 1 | M | 25.2g |  |
| V22 |  | Left 2 |  |  |  |
| V23 |  | Right 1 |  |  |  |
| V24 |  | Right 2 |  |  |  |
| V25 | M7 | Left 1 | M | 25.5g |  |
| V26 |  | Left 2 |  |  |  |
| V27 |  | Right 1 |  |  |  |
| V28 |  | Right 2 |  |  |  |
| V29 | M8 | Left 1 | M | 21.5g |  |
| V30 |  | Left 2 |  |  |  |
| V31 |  | Right 1 |  |  |  |
| V32 |  | Right 2 |  |  |  |
| V33 | M9 | Left 1 | M | 27.8g |  |
| V34 |  | Left 2 |  |  |  |
| V35 |  | Right 1 |  |  |  |
| V36 |  | Right 2 |  |  |  |
| V37 | M10 | Left 1 | M | 25.9g |  |
| V38 |  | Left 2 |  |  |  |
| V39 |  | Right 1 |  |  |  |
| V40 |  | Right 2 |  |  |  |
