# Supplementary material for: Scratch-AID, a deep learning-based system for automatic detection of mouse scratching behavior with high accuracy
Source: eLife. 2022 Dec 8;11:e84042. doi: 10.7554/eLife.84042 (PMC9762698; doi:10.7554/eLife.84042)
Supplement: Supplementary file 2. [file elife-84042-supp2.docx]

**Supplementary file 2. Scratching behavior summary in the 40 training and test videos (reference annotation)**

| Video No. | Total scratching train | Total scratching frames | Total scratching time (s) | Scratching time percentage (%) |
| --- | --- | --- | --- | --- |
| V1 | 13 | 9785 | 326 | 27.2 |
| V2 | 19 | 7511 | 250 | 20.9 |
| V3 | 27 | 10359 | 345 | 28.8 |
| V4 | 28 | 11022 | 367 | 30.6 |
| V5 | 47 | 8932 | 298 | 24.8 |
| V6 | 57 | 12519 | 417 | 34.8 |
| V7 | 47 | 8532 | 284 | 23.7 |
| V8 | 37 | 9790 | 326 | 27.2 |
| V9 | 24 | 6349 | 212 | 17.6 |
| V10 | 36 | 7964 | 265 | 22.1 |
| V11 | 43 | 10615 | 354 | 29.5 |
| V12 | 66 | 7877 | 263 | 21.9 |
| V13 | 1 | 371 | 12 | 1.0 |
| V14 | 16 | 8381 | 279 | 23.3 |
| V15 | 34 | 8935 | 298 | 24.8 |
| V16 | 15 | 7356 | 245 | 20.4 |
| V17 | 11 | 4123 | 137 | 11.5 |
| V18 | 37 | 11965 | 399 | 33.2 |
| V19 | 20 | 6000 | 200 | 16.7 |
| V20 | 26 | 10794 | 360 | 30.0 |
| V21 | 20 | 8226 | 274 | 22.8 |
| V22 | 21 | 10071 | 336 | 28.0 |
| V23 | 47 | 12872 | 429 | 35.8 |
| V24 | 23 | 7306 | 244 | 20.3 |
| V25 | 11 | 4137 | 138 | 11.5 |
| V26 | 25 | 7152 | 238 | 19.9 |
| V27 | 29 | 10103 | 337 | 28.1 |
| V28 | 31 | 10424 | 347 | 29.0 |
| V29 | 7 | 2402 | 80 | 6.7 |
| V30 | 11 | 7559 | 252 | 21.0 |
| V31 | 22 | 5559 | 185 | 15.4 |
| V32 | 24 | 7595 | 253 | 21.1 |
| V33 | 19 | 6900 | 230 | 19.2 |
| V34 | 40 | 7525 | 251 | 20.9 |
| V35 | 19 | 4418 | 147 | 12.3 |
| V36 | 44 | 7538 | 251 | 20.9 |
| V37 | 28 | 8005 | 267 | 22.2 |
| V38 | 33 | 8078 | 269 | 22.4 |
| V39 | 26 | 5998 | 200 | 16.7 |
| V40 | 50 | 6168 | 206 | 17.1 |
